# Development and validation of a 70K SNP genotyping array for Atlantic halibut (*Hippoglossus hippoglossus)*

**DOI:** 10.1186/s12864-025-12128-1

**Published:** 2025-10-16

**Authors:** Valentina Krivenjeva Sinani, Robert C. Wilson, Torfinn Nome, Ujjwal Acharya, Elisabeth Kommisrud, Marte Wetten, Matthew P. Kent

**Affiliations:** 1 Department of Biotechnology, CRESCO, Centre for Embryology and Healthy Development, University of Inland Norway, Hamar, Norway; 2 Department of Animal and Aquacultural Sciences, Centre for Integrative Genetics, Faculty of Biosciences, Norwegian University of Life Sciences, Ås, Norway; 3 Cryogenetics AS, Hamar, Norway; 4 Aninova AS, Hamar, Norway; 5 Natural History Museum, University of Oslo, Oslo, Norway

**Keywords:** Hippoglossus hippoglossus, SNP-array, Diagnostic gender determination

## Abstract

**Background:**

The analysis of single nucleotide polymorphism (SNP) genotypes which capture genome-wide polymorphic variation can be used to improve the accuracy of selection in breeding programs and reveal the genetic architecture of quantitative traits. Mid-density SNP genotyping arrays containing > 60 K SNP markers have been developed for numerous production species and have been used to enable enhanced breeding progress. Despite recent genomic advances, Atlantic halibut lacks mid-density genotyping tools, limiting the application of genomic selection and high-resolution association studies in this species. The evolving Atlantic halibut (*Hippoglossus hippoglossus*) industry is also interested in genetically informed selection of breeding candidates to facilitate genetic improvement. The present study reports the development of the first mid-density SNP-array for use in Atlantic halibut and describes its performance in samples from different breeding populations.

**Results:**

Variant discovery was performed using whole genome sequencing (WGS) data from 40 individuals and resulted in the detection of more than 8 million small genetic variants including 6,224,375 SNPs and 2,131,436 insertion-deletion variants (INDELS). An Affymetrix Axiom custom array containing 70,824 assays (including 697 for determining genetic gender) was created and used to genotype 1152 Atlantic halibut samples. Assays for 59,491 SNP loci (84% of the array total) performed well and generated reliable genotypes, with an average SNP call rate of 99.64% and genotype concordance of 99.0% with whole-genome sequencing data. Of the gender-specific loci, 133 SNP assays could be used to assign genetic gender.

**Conclusions:**

This article describes the first mid-density SNP genotyping array for Atlantic halibut. It contains nearly 60 K robust assays for genome-wide dispersed SNP loci, along with those suitable for determining genetic gender. The array is amenable for use as a platform for high-resolution genetics research into traits of evolutionary and economic importance and the results have practical relevance for genomic selection in Atlantic halibut.

**Supplementary Information:**

The online version contains supplementary material available at 10.1186/s12864-025-12128-1.

## Background

Atlantic halibut (*Hippoglossus hippoglossus*) belongs to the Pleuronectidae family of flounders and is distributed throughout the northern part of the North Atlantic Ocean and in parts of the Arctic Ocean. Being the largest species in this family, Atlantic halibut is a highly valued food source that has become scarce due to overfishing and subsequent stock collapses [1]. To support informed conservation, stock management, and diversity evaluation for this valuable marine species, thereby promoting sustainable aquaculture, reliable genetic markers must be identified and developed.

Over the last decades, the advancement of new molecular methods has given new impetus to aquaculture in genetic and genomic research [2]. Genomic tools can be very beneficial in facilitating sustainable genetic improvement [3]. SNP genotyping arrays have been developed for several aquaculture species including Atlantic salmon which was among the first to have a high-density SNP array validated and widely applied in genomic selection programs [4]. Since then, arrays have been created for diverse species including Nile tilapia [5], European seabass and gilthead seabream [6], and pufferfish [7], where they have been used for applications such as identifying sex-determining loci, mapping quantitative trait loci (QTLs), and enabling selective breeding. A recent broad review also highlights the rapid expansion of SNP arrays across aquaculture, underscoring their importance for genomic selection, population genomics, and fisheries management [8].

Compared to other fish species like salmon, rainbow trout and common carp where genomic selection is being practiced, halibut has been slower to adopt genome-based selection tools largely due to a lack of genomic resources such as reference genomes, SNP arrays, and linkage maps. Several genomic tools have recently been developed for Atlantic halibut, including the first genome assembly [9] and a 4 K SNP array [10] that support future large-scale genetic analysis. A recent study [11] generated a genome assembly for Atlantic halibut and identified *gsdf* as the sex determining gene in this species.

Slow growth, high mortality, weaning, diet, pigmentation, reovirus infection, and other challenges affect halibut production. And there is evidence from other species such as salmon [4, 12], tilapia [5, 13], rainbow trout [14, 15], turbot [16], carp [17, 18], seabass and seabream [6, 19] that use of genomic tools has contributed to solving these challenges.

In this study, the aim was to identify genome-wide SNP markers in Atlantic halibut for the purpose of generating a 70 K SNP genotyping array. SNPs were detected by mapping whole genome sequencing data from forty selected fish to the reference genome established by Edvardsen et al. [11] prior to filtering for inclusion in the final 70 K Affymetrix Axiom array. Male-specific polymorphisms upstream of *gsdf* were also included as input in the generation of this array for the purpose of validating their use in determining the genetic sex of the halibut individuals analyzed in this study.

## Materials and methods

### Sampling and whole-genome sequencing

Fin-clips from 40 sexually typed broodstock fish, 20 males and 20 females, but otherwise exhibiting a broad range of other phenotypes concerning pigmentation, weight, age (range ca. 5–10 years) within each sexed group, were collected at five different commercial halibut production facilities and stored in 70% ethanol; with few exceptions, these samples were taken non-lethally. One Scottish producer contributed 7 samples, while four Norwegian producers contributed 13, 10, 7 and 3 samples, respectively. Genomic DNA was extracted from 20 mg fin tissue by first incubating with 20 µl Proteinase K (20 mg/ml) in 230 µl Buffer ATL (Qiagen) overnight at 56 °C. DNA was isolated from the resulting lysates the next day using the Maxwell^®^ 16 Tissue DNA Purification Kit (Promega) and the Maxwell^®^ 16 Instrument (Promega) according to manufacturer’s instructions. Purified DNA was prepared for sequencing by a commercial provider (Novogene UK) using the NEBNext^®^ DNA Library Prep Kit (New England BioLabs, US) before being sequenced on an Illumina NovaSeq 6000 using an S4 flow cell to generate 2 × 150 base paired-end reads.

### SNP detection and array locus filtering

Raw data from 40 whole genome sequenced samples were aligned to the reference reported by Edvardsen et al. [11] using bwa-mem2 (version 2.1, 10.1109/IPDPS.2019.00041) with default parameters; duplicate reads were marked using Samblaster (version 0.1.26). Samtools (version 1.9) was used to output CRAM files, after piping all reads through the “samtools fixmate” command. SNP calling was performed using DeepVariant (version 1.1.0; Poplin et al. 2018) with default WGS parameters (“--model_type = WGS”). Genomic variant call files (gVCF) for each sample were merged from DeepVariant into a single VCF file by GLnexus (version 1.2.2, 10.1101/343970) using the “—config DeepVariantWGS” parameter. The VCF file was furthered filtered to remove SNPs with minor allele frequencies (MAF) < 0.1, and/or read depths > 29 or < 6 using BCFtools (version 1.10.2, 10.1093/gigascience/giab008) with following parameters: “-i ‘AF > 0.1 & AF < 0.9 & FMT/DP > 5 & FMT/DP < 30’”, and allowing only bi-allelic SNPs excluding W and S which require twice as many probes to genotype using the Axiom chemistry. Finally, ChipChop (https://github.com/haraldgrove/chipchop*)* was used to identify and select a set of SNPs distributed along a scaffold and spaced at 3 Kb intervals.

This resulted in a list of 138 K candidate SNPs distributed over the 24 larger scaffolds encompassing 97.6% of the IMR_hipHip.v1 genome assembly, and 1366 candidate SNPs distributed over 162 unplaced scaffolds representing the remaining 2.4% of the genome. Since the total SNP number still exceeded the array’s theoretical maximum capacity of approximately 75 K, we further trimmed these lists and removed SNPs with minor allele frequencies (MAF) = 0.5, and those closer than 3150 bp to their nearest preceding SNP, concluding with a list of 91,113 markers. This list was further enriched with loci that could potentially be used for gender determination. Specifically, (a) 245 SNP polymorphisms located within the 3.5 Kb *gsdf* gene region, (b) 59 SNPs identified in our WGS data and that, from Edvardsen et al. [11], behave in a gender-specific manner (homozygotes in females and heterozygous in males), and (c) 334 non-polymorphic loci located around (+/- 100 bp) both the upstream and downstream breakpoint junctions between the two transposable elements (chr13:8503901–8505128; 1,227 bp, and chr13:8509688–8509767; 79 bp) coinciding with the *gsdf* locus in males as reported by Edvardsen et al. [11]. The combined list of 91,423 SNPs was submitted for in silico validation by the Affymetrix Bioinformatic Service. This evaluation pipeline predicts the performance of SNPs and calculates a conversion probability value (p-convert value: representing the probability of a given SNP converting to a reliable SNP assay on the Axiom array system) using various criteria including binding energy, GC content, and the expected degree of non-specific hybridization to multiple genomic regions. Based on the p-convert values, they classify the SNPs into different categories: recommended, neutral, not recommended. Removal of “not recommended” SNPs and some final filtering based on inter-SNP distance resulted in a final list of 70,824 SNPs including 638 potentially suitable for gender determination and 70,141 that are distributed genome wide.

### SNP genotyping

Fin clips from 1152 fish were provided by commercial halibut producers, and DNA was extracted using the Maxwell^®^ 16 Tissue DNA Purification Kit (Promega) and the Maxwell^®^ 16 Instrument (Promega) according to manufacturer’s instructions. After quantification and analysis of purity, DNA was genotyped on the new CryoHh01 array at the Center for Integrative Genetics (CIGENE, NMBU) in Norway. Individual samples were considered to have been successfully genotyped if they passed the Axiom Best Practices Workflow with default settings (sample Dish QC ≥ 0.82, QC call rate ≥ 97; SNP call-rate cutoff ≥ 97).

### Linkage disequilibrium estimation

Pearson’s squared correlation coefficient (r^2^) statistic was used to estimate the Linkage disequilibrium (LD) between each pair of SNP markers, which is less sensitive to allelic frequencies and more appropriate for biallelic markers. Pair-wise LD as r^2^ values based on the formula of Hill and Robertson (1968) were estimated using Plink v1.09 [20] using to the parameters listed in Table S2.

In order to estimate correlations between all the pairs of SNPs within each chromosome independently, the parameter –inter-chr was used in conjugation with a ld-window set to zero.

Bins of 100 Kb were created for each SNP pair based on their physical distance. And the decay and extent of the LD were calculated. LD decay curves for SNP pairs were determined as the average r^2^ within each bin, up to a distance of 10 Mb. Average r^2^ per chromosome was calculated, sorting SNPs pairs into 10 bins according to an increasing average distance. The distance used was from 0 to 0.99 Mb and from 9 to 10 Mb (in the first and last bin, respectively), between SNPs pairs on each chromosome.

## Results

### Whole genome sequencing, SNP filtering and array design

An average of 17.1 Gb (SD 0.48 Gb) raw sequence data was generated for each sample, equivalent to approximately 23x coverage assuming a haploid genome size of 750 Mb. The initial variant detection revealed > 8 million variants across the 40 samples including 6,224,375 SNPs and 2,131,436 indels. Stringent filtering was used to reduce this to a list of 70,824 loci including 697 potentially informative for genetic sex.

### Genotyping performance of the SNP array

**Fig. 1:**
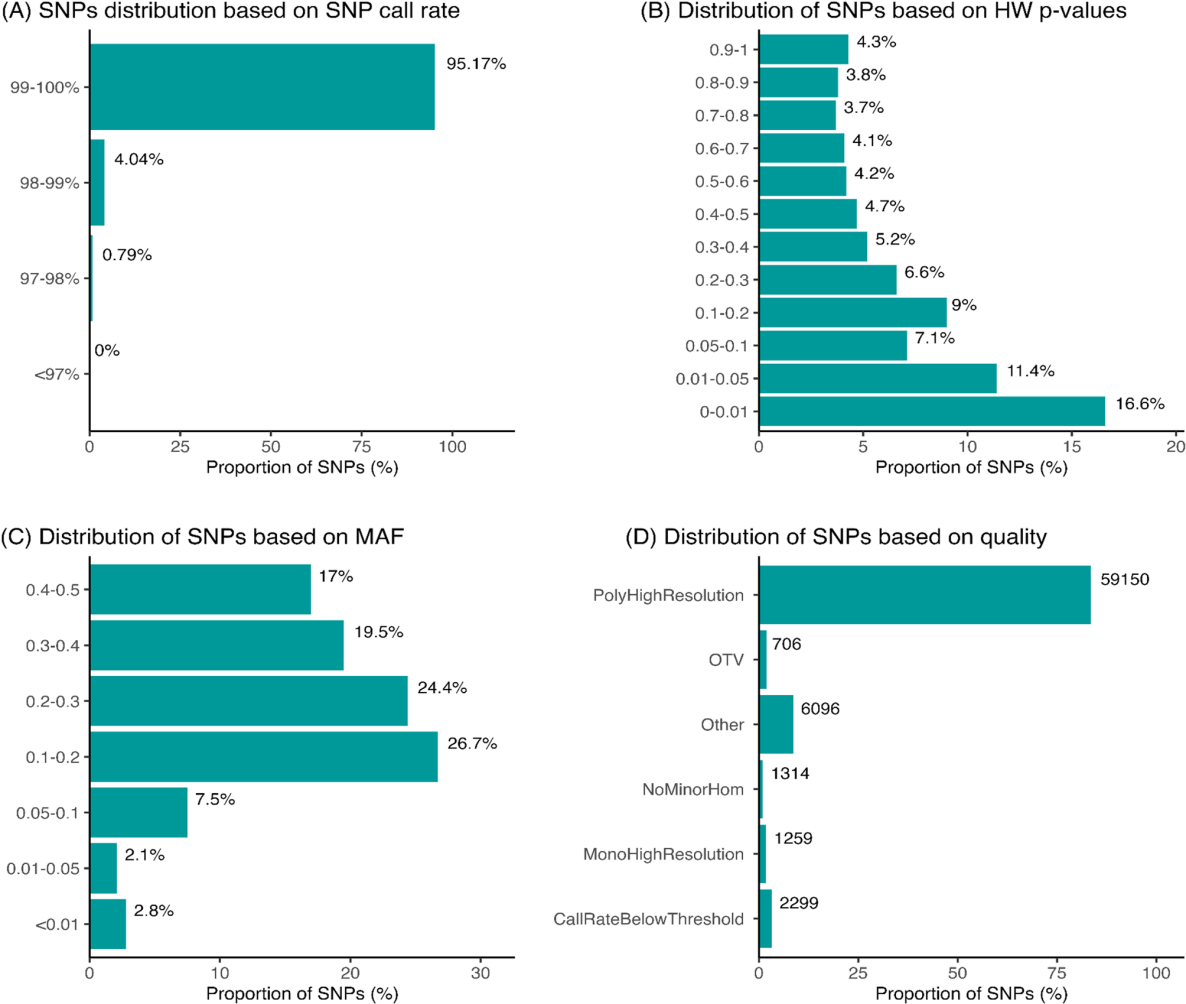
Summary of the SNP metrics based on 70,824 SNPs on 1138 samples after being subjected to sample call rate in Best Practices Workflow of Axiom Analysis Suite software. (**A**) SNPs distribution based on SNP call rate. (**B**) Distribution of SNPs based on Hardy-Weinberg (HW) *p*-values. (**C**) Distribution of SNPs based on minor allele frequency (MAF). (**D**) Distribution of SNPs based on quality across the six defined classes. The value on each bar represents the number of SNPs

A total of 1152 samples were genotyped on the CryoHh01 array; 14 samples failed the quality control call-rate threshold of 97% and were excluded from analysis; the remaining 1138 samples had an average call rate of 99.64%. Around 17% or 11,725 SNPs (p-value < 0.01) were found to depart from Hardy-Weinberg equation. Furthermore, 95% of the SNPs had minor allele frequencies (MAF) > 0.05, and 5% had MAF < 0.05. The Axiom Analysis Suite (AAS) software [21] performs automated SNP classification based on multiple parameters capturing aspects of assay performance. Figure 1 shows more specific information about the sample and SNP statistics.

### Validation of array performance

To further assess the quality and reliability of the array SNP genotypes obtained, we compared them with those reported in the variant calls file (vcf) generated from the whole genome sequencing data for 36 of the 40 individuals used for SNP detection; the 4 excluded individuals had array genotyping call rates less than 80%. For this comparison, we included genotypes only from genome-wide distributed SNPs (*n* = 70490) and did not include data from the 334 non-polymorphic assays related to transposable element insertion-based gender determination. The best performing SNP category: PHR, represents 84% of SNPs on the array (*n* = 58,918), and 88.2% of these (51,970) showed 100% genotype concordance between array and sequencing data (see Table 1). The 11.8% of PHR SNPs that showed discordance at a frequency of 2.9% (i.e., on average the disagreement was restricted to one of the 36 individuals being compared), and revealed an overall concordance (total possible agreeing genotypes/total discordant genotypes) of 99.0% for the PHR SNP class, with the remaining SNP classes displaying variable concordance. The SNPs showing the poorest concordance were those characterized as Other, Off Target Variants (OTV) and Monomorphic High Resolution (MHR) which are all likely unsuitable since their performance on the array suggests confounding effects caused by nearby, undetected mutations, or that they are false-positive loci. The categories No-Minor Homozygous (NMH) and Call-Rate below Threshold (CRbT) include potentially amenable markers and show concordances > 91%.

**Table 1:** Concordancy between array and sequence assigned genotypes for genome distributed SNPs. SNP assays were grouped by axiom analysis suite (AAS) into polymorphic high resolution (PHR), no-minor homozygote (NMH), monomorphic high-resolution (MHR), call-rate below threshold (CRbT), off-target variant (OTV) and Other. Within each group the table reported the proportion included on the array, their concordance and discordance with genotypes from 36 samples, and their overall concordance as a percentage

SNP category	# on array	% on array	# showing 100% concordance	# showing discordancy	Average discordancy	Overall % concordance
PHR	58,918	84.0	52,064	6854	2.9	99.0
NMH	622	0.9	434	188	10.1	91.4
MHR	1085	1.5	0	1085	16.1	55.1
CRbT	2284	3.3	879	1405	5.1	91.2
OTV	1301	1.9	7	1294	15.2	58.0
Other	5931	8.5	972	4959	11.3	73.7
Total	70,141		54,356	15,785		

A second assessment of SNP array performance was made possible by independently genotyping a small number of samples (*n* = 9) twice on the array. Considering data from all 70,824 SNP assays, the average number of inconsistencies between sample pairs was 1698 (2.4%); these inconsistencies are predominantly resulting from instances where, for a pair of genotypes, one is missing (82% of cases) and less commonly from discordance between reported genotypes (18%). When repeating this analysis but restricting it to the robust PHR and NMH SNP assays (*n* = 59,856), the average number of discordances dropped to 232 (< 0.4%) indicating that most of the discrepancies are within non-PHR SNP categories.

#### Estimating linkage disequilibrium

**Fig. 2:**
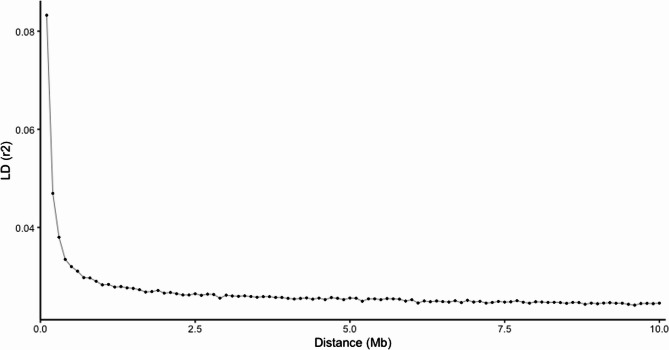
Decay of average LD (*r*^2^) over distance among SNPs in Atlantic halibut (Hippoglossus hippoglossus) populations. The black dots show the average r^2^ within each bin, with a sliding window of 100 Kb

For estimating the decay of linkage disequilibrium as a function of physical distance, SNP pairs were classified into 100 Kb bins and mean r^2^ values were computed for each bin (Fig. 2). LD decreases steadily when the physical distance between markers rises, as found in other species [22, 23].

For SNPs less than 0.1 Mb apart, a maximum average LD of 0.083 was estimated. This value declines quickly at the distance 0.2 Mb with average LD of 0.047. The lowest estimate of average LD of 0.017 was found at a distance of 10 Mb. Figure 2 clearly shows that there is almost no LD between SNPs separated by large physical distances (Mb scale). Therefore, we tested with a shorter physical distance of 20 kb (Supplementary Figure S1) and it shows that LD between markers at a physical distance below about 0.5 Mb is significantly greater.

**Fig. 3:**
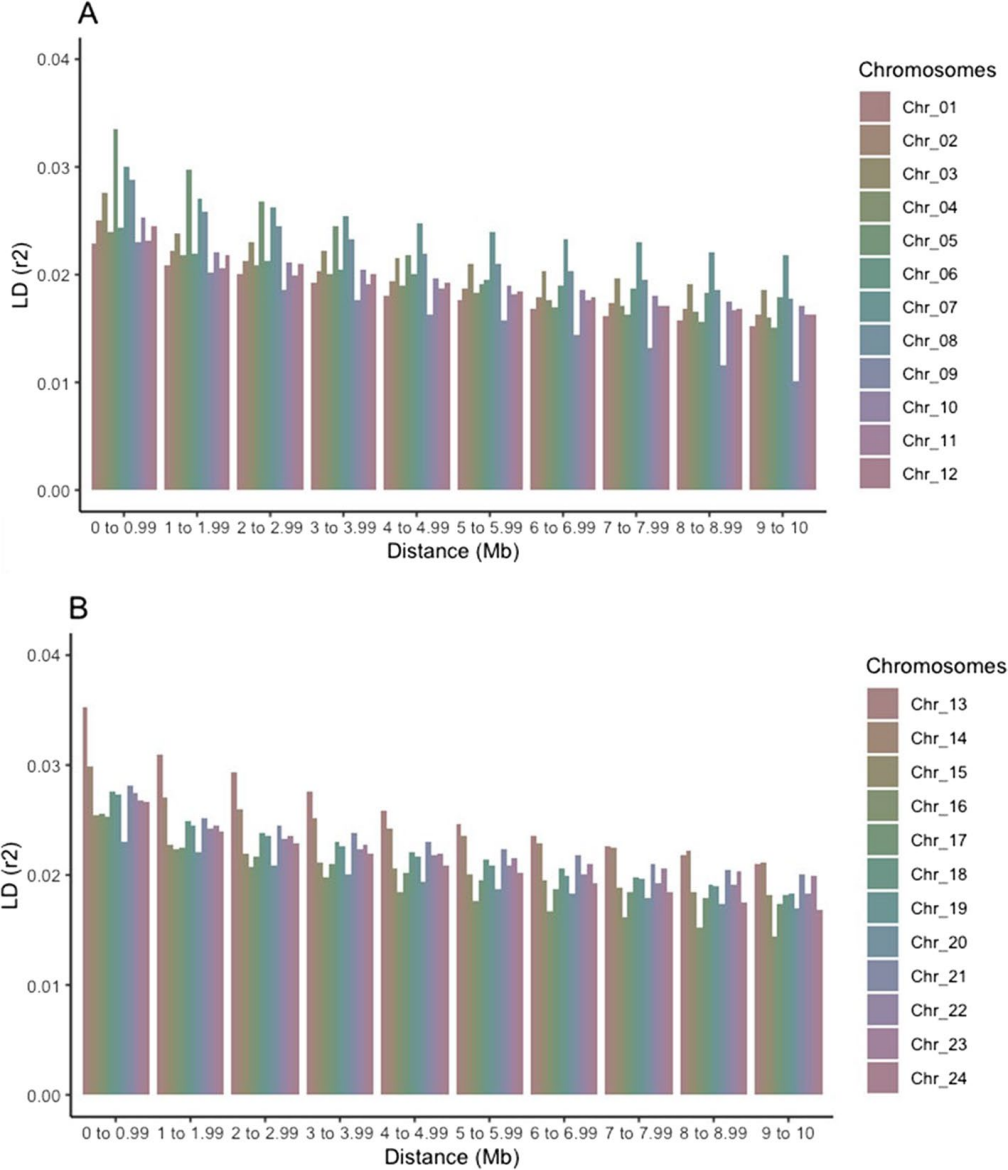
Linkage disequilibrium analysis along the 24 chromosomes of Atlantic halibut. Average values of LD measured as *r*^2^ per chromosome, according to distances between SNPs. Estimated values are shown from chromosome 1 to 12 (Chr_1 to Chr_12) in panel **A** and from chromosome 13 to chromosome 24 in panel **B**

Comparison of average LD at different distance bins for each chromosome shows higher variation at closer distance bins (Fig. 3). Lower estimates of LD (< 0.023) were found in chromosomes 1, 9, 16 and 20, while higher levels of LD (> 0.025) were estimated for chromosomes 3, 5, 7, 8, 13 and 14. Furthermore, average *r*^2^ values < 0.02 were estimated for all chromosomes, except for chromosome 3, 7, 8, 13, 14, 21 and 23 at distances greater than 7 Mb.

#### Sex probes analysis and validation

We included 638 putatively sex-linked probes on the array, of which 604 performed satisfactorily and gave usable data. Three categories of probe were included, (i) probes for sex-linked SNPs from RNA-seq, (ii) probes interrogating SNPs detected within the *gsdf* gene, and (iii) probes testing for the presence of inserted TE sequences. We examined their genotypes in 154 individuals that were phenotypically sexed by the producers as 114 females, 22 males and 18 neomales (genetic females but phenotypic males; please consult Supplementary Figure S2 for an overview the workflow for validation of the three probe categories).

**Fig. 4:**
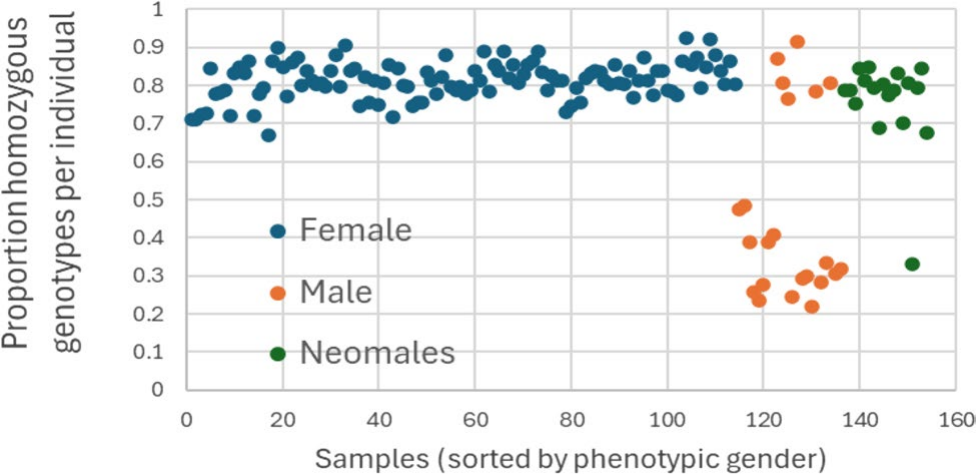
Per sample, the proportion of homozygous genotypes for 59 SNPs previously identified within coding sequence as segregating in males and females

SNPs known to be homozygous in females and heterozygous in males are arguably those most likely to be simple to interpret and gender-informative. In their paper, Edvardsen et al. [11] used expression of *gsdf* as a proxy for gender and were able to categorize samples, for which they had RNA-seq data, as females (*n* = 5) or males (*n* = 7). Thereafter they identified 65 SNPs that were fixed (homozygous) in females and variable (heterozygous) in males in transcript sequences aligning at positions distributed widely across chromosome 13. We verified 59 of these polymorphisms in our whole-genome sequencing (WGS) SNP data (*n* = 40) and included probes for these on the SNP array. Calculated across all 59 SNPs, Fig. 4 shows the proportion of homozygous genotypes per sample in phenotypic females, males and neomales. As expected, samples identified as female exhibited high homozygosity while phenotypic male samples showed significantly lower homozygosity. Interestingly, 6 males displayed homozygosity consistent with them being female, while one neomale behaved as a true genetic male raising the possibility that phenotypic gender determination was imperfect.

Because there are typically fewer SNPs in protein-coding genes than non-coding DNA [21] and to increase the robustness of gender assignment, we explored the homozygosity of SNPs detected in our WGS data from 40 individuals across the annotated *gsdf* sequence (chr13:8,506,700-8,510,213). This revealed 338 polymorphisms, whereof 211 were amenable for probe design and, after genotyping, exceeded minimum acceptable quality performance. Collectively, the per-sample proportion of homozygosity showed a similar trend as in Fig. 4 where SNPs were pre-selected based on gender but was less convincing (see Supplementary Figure S3), however by exploring the performance of individual markers it was possible to identify 42 polymorphisms that showed extreme differences in homozygosity.

**Fig. 5:**
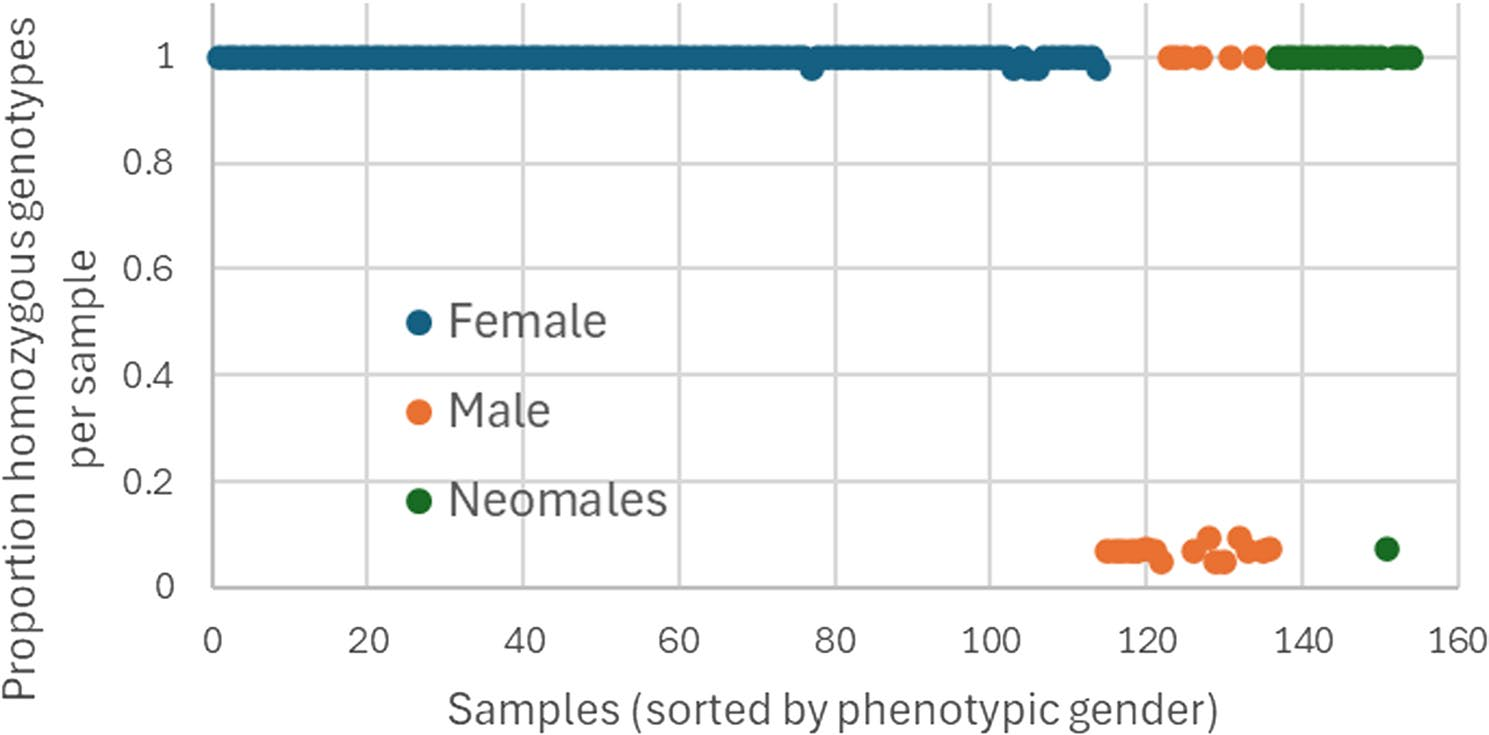
Per sample, the proportion of homozygous genotypes for 42 SNPs identified within the *gsdf* gene sequence

Again, the results from these 42 SNPs highlight 6 samples classified phenotypically as male that seem to behave as genetic females and one neomale that seems to be genetically male (Fig. 5). The inconsistent samples have the same identities in both RNA- and *gsdf* WGS- sourced SNP analyses.

The final class of potentially gender informative probes (334) were designed to interrogate non-polymorphic bases 100 bp upstream and downstream of the breakpoints flanking the insertion of each of the two Gypsy-like transposable elements (TE) reported by Edvardsen et al. [11]. Because the TE is present at multiple locations in the genome, it is only possible to deduce its presence or absence at this specific location by designing probes that interrogate its boundaries with unique sequence. The axiom technology requires that for each genomic target, an immobilized capture oligonucleotide probe is ligated to a labelled probe after hybridizing perfectly next to each other along a complementary genomic template forming a 70-mer. A non-complementary template will not facilitate efficient ligation, and no signal will be detected from the immobilized probe. These “TE-breakpoint” probes would, in theory, only generate signals in males, and no signal would be detected in females due to imperfect hybridization.

**Fig. 6:**
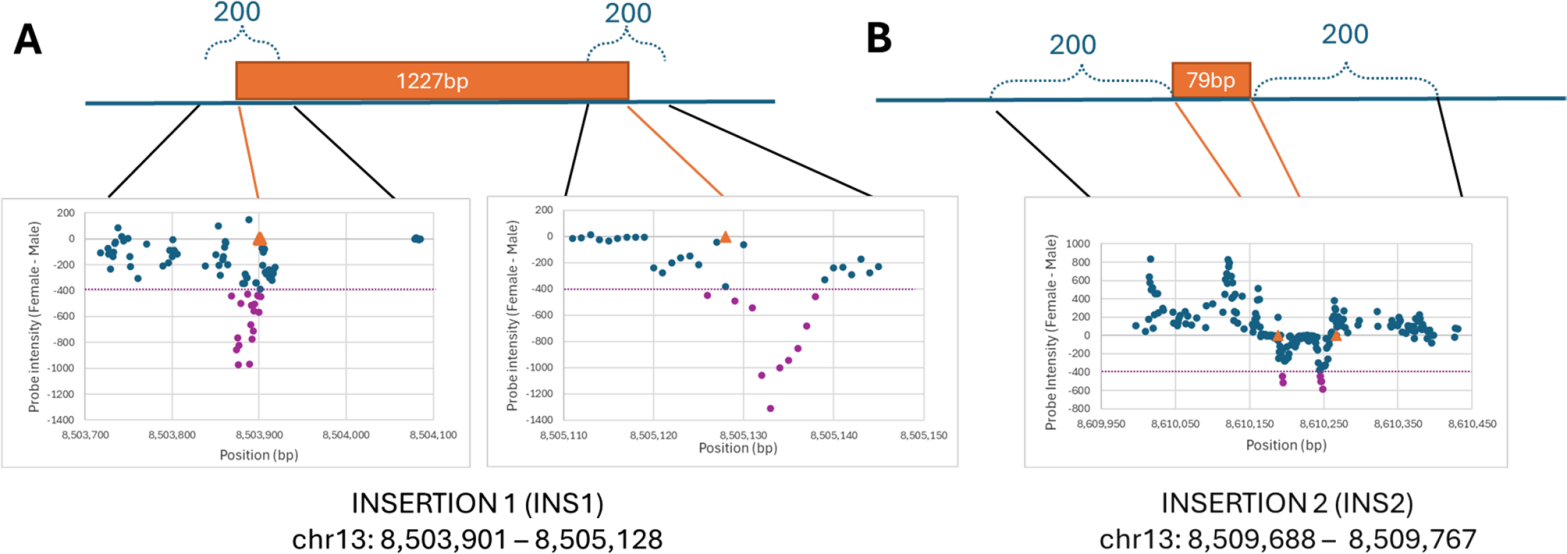
Schematic illustration of probe annealing location relative to reported TE insertion sites. X-axis includes position of the array-bound 35-mer probes 5’-nucleotide within Chr13, while Y-axis reports the difference in probe signal intensity (average female signal - average male signal). Probes in purple fall below an arbitrary threshold where average intensity in females is at least 400 less than males. Orange triangles pinpoint the predicted insertion breakpoints

Insertion 1 (INS1; 1227 bp) is upstream of *gsdf* in Atlantic halibut and speculated to act as a rogue promoter driving gene expression. We observed a noticeable drop in signal for those capture probes hybridizing near the speculated upstream and downstream INS1 breakpoints (Fig. 6A).

Insertion 2 (INS2) lies within the 3’-UTR of *gsdf* and is 79 bp long. As for INS1, probes were developed using Axiom design software to interrogate genomic DNA around and including the upstream and downstream TE breakpoints (Fig. 6B); binding sites of selected oligonucleotides (individual sex probe sequences) on chromosome 13(LG13) of Atlantic halibut are listed in Supplementary Table S1. As before, a negative signal represents reduced signal in females which is the expected outcome if the TE is absent in females. A drop in female signal is observed in the approximate appropriate locations for the TE, however there is also an elevated female signal seen in probes 3’ to the TE breakpoint which can indicate other gender specific structural differences, for example sequence copy-number variations, or interfering SNP polymorphisms. This emphasizes the fact that only a subset of the 334 probes behave in the expected manner, selecting probes at the putative TE breakpoints from both INS1 and INS2 with an arbitrarily chosen intensity difference of 400 (i.e., average female - male signal intensity ≥ −400) returns 34 probes, calculating the average intensity for these in males, females and neomales shows a clear differentiation and again reveals a consistent difference between phenotyping and genotypic gender.

Using this array to determine genetic sex in Atlantic halibut seems plausible. The probes indicating the presence of TE-insertions at the *gsdf* locus on chromosome 13 are essentially serving to genotype two specific structural variants (SVs). Variation in the exact location of SV breakpoints in different individuals can impact the performance of these probes and confound sex-genotyping. Similarly, this limited validation set (154 samples) may display allelic bias making certain SNPs less informative about samples from other populations. Therefore, we recommend that use of the array data for gender determination includes collective and critical analysis of all of the most strongly sex-linked probes, specifically the 59 and 42 polymorphic loci detected from RNAseq and whole genome GSDF analysis, and the 32 TE interrogating probes.

## Discussion

The development of a mid-density SNP genotyping array for Atlantic halibut represents a significant advancement in genomic research for this species. The CryoHh01 array, with approximately 70 K SNPs, provides a robust tool for genetic studies, genomic selection, and sex determination. The validation of this array demonstrated high genotyping accuracy, with an average call rate of 99.64% and strong SNP concordance with whole-genome sequencing data. The overall performance of the CryoHh01 array is in line with other teleost SNP arrays: the MedFish (~ 60 K) reported high conversion in European seabass and gilthead seabream with repeatability ~ 99.4–99.7% [6], the 58 K Nile tilapia array retained ~ 74–75% PolyHighResolution SNPs with stringent sample pass rates [5], and the 57 K rainbow trout array showed high validation of polymorphic assays [15].

The performance of the array was validated by genotyping 1152 individuals, revealing that 84% of the SNPs provided reliable genotype calls. The existing 4 K SNP panel for Atlantic halibut described by Weise et al. [10] was designed to maximize power for kinship analysis, with marker density intentionally higher in the sex-determining region and in a chromosomal inversion. This panel also enables non-lethal sex determination. Our 70 K SNP array complements this resource by providing genome-wide coverage of higher resolution suitable for applications such as fine-scale mapping, genomic selection, high-resolution population genetics, and phenotype association analyses, including sex determination. The SNP densities offered by RAD sequencing approaches to date, or even the existing SNP panel [10] are not sufficient to capture population-wide linkage disequilibrium to enable fully effective genome-wide association studies.

Around 17% of the SNPs were found to depart from Hardy-Weinberg Equilibrium (HWE) (*p* < 0.01). Departure from HWE may occur due to a variety of causes, including purifying selection, population substructure, inbreeding or copy number variations [24]. As we know from the producers, bred halibut has not undergone many generations of selection, but the base population was established with very few parents which could perhaps have generated a founder effect.

The estimation of linkage disequilibrium between molecular markers within a population is critical when establishing the minimum number of markers required for association studies, genomic selection, and inferring historical events influencing different populations. To our knowledge, this is the first study characterizing whole-genome LD in Atlantic halibut populations using a mid-density SNP array. Studying LD patterns broadens our understanding of demographic dynamics within the population. Biological factors such as recombination and mutation in conjunction with genetic drift, admixture and effective population size are important variables determining patterns of LD. Variations in LD among populations and genomic regions is therefore highly important and frequently reported [5, 22, 25]. In brief, LD in our broodstock decays from moderate at short distances to low beyond a few megabases—consistent with farmed salmonids (e.g., coho)—and supports a view that the present ~ 70 K density is adequate for GWAS and within-population genomic selection [22].

LD generally declines with increasing physical distance between SNPs, but the way this pattern emerges can depend heavily on how the data is grouped. When using broader bins, the overall trend of decay becomes apparent, with LD approaching low levels at longer distances. However, looking at smaller distance intervals can highlight stronger associations between nearby markers that might otherwise be overlooked. This gradual shift in LD likely reflects the influence of recombination and other genomic factors that shape the extent of non-random associations across the genome. Comparable values of LD for farmed Norwegian Atlantic salmon populations have been reported [25] However, the low level of LD found in the halibut populations analyzed in this study is consistent with their more diverse origins, considering the fact that the broodstock populations of halibut have historically been restocked with wild fish. Furthermore, the halibut populations were established using fish from several locations, which likely favored greater genetic diversity.

Atlantic halibut are slow growing and show late onset of sexual maturation and because of this it is desirable to know the genetic gender of individuals in a breeding-candidate population without the need for physical phenotyping. Teleost fish display diverse, plastic sex determination systems but a recent study [11] developed a high-quality genome assembly of Atlantic Halibut (*Hippoglossus hippoglossus*) and proposed gonadal somatic cell derived factor (*gsdf*) as the gene encoding the sex determining factor, they also identified specific SNP loci and two Gypsy-like transposable elements (TEs) that show evidence of being associated with genetic sex. In our study, the CryoHh01 array included multiple assays designed to interrogate loci segregating in males and females and was able to clearly differentiate males and females based on homozygosity, even revealing a likely phenotypic misassignment of 6 females and 1 neomale. A total of 59 loci previously reported in RNA-seq data [11], and 42 newly identified loci in *gsdf* contributed to this analysis. Their usefulness as gender discriminators is based on analyzing their collective average homozygosity, but with further genotyping of phenotypically assigned samples, it may be possible to refine and reduce this list. Probes hybridizing to putative breakpoints flanking the insertion of each of the two Gypsy-like transposable elements (TE) also showed potential for assigning gender although this was based on signal intensity rather than a nucleotide genotype where the hypothesis is that a drop in intensity reflects reduced hybridization efficiency due to the presence of a TE. The upstream breakpoint of INS1 seems particularly informative with multiple overlapping probes coinciding with the reported breakpoint and demonstrating a robust negative signal, however, a lack of knowledge about the precise breakpoints in each of the 154 phenotyped fish makes it difficult to unambiguously conclude which of these probes can reliably genotype the insertions.

Areas for future work include (i) quantifying genomic selection accuracy for key traits—growth, survival, weaning success, pigmentation, and viral disease resistance—together with assessment of cross-population portability and potential ascertainment bias; (ii) performing genome-wide association studies on these traits to detect QTL and iteratively refreshing array content by prioritizing GWAS-flagged SNPs and adding informative lower-MAF variants to maintain tagging performance; and (iii) increasing genetic prediction accuracy by enlarging and balancing the training population, refining marker quality control, and benchmarking alternative prediction models (e.g., GBLUP vs. Bayesian, including multi-trait and environment-specific models).

## Conclusion

We present a novel SNP array suitable for genotyping Atlantic halibut. The array interrogates ~ 70 K SNP polymorphisms distributed across the genome and includes informative assays for genetic sex identification. As well as being a useful tool for breeders seeking to control pedigrees or apply marker assisted selection (MAS) or genomic selection (GS), the array can be used for performing GWAS based QTL studies and be used to map and monitor wild populations.

## Supplementary Information

Supplementary Material 1. Figure S1. Decay of average LD (r^2^) over distance among SNPs in Atlantic halibut (*Hippoglossus hippoglossus*) population. The black dots show the average r^2^ within each bin, with a sliding window 20 Kb. Table S1. List of SNP probes tested for sex identification. Results showing binding sites of 67 oligonucleotides (individual sex probe sequences) on chromosome 13(LG13) of Atlantic halibut; fwd: forward. Table S2. List of SNP Parameters used in PLINK to run LD. Figure S2. Diagram summarizing the workflow of the sex probes analysis and validation. Figure S3. Proportion of homozygosity for 211 SNPs across the gsdf region per individual. The trend is for males to display lower homozygosity, but the contrast is not strong.

## Data Availability

The reference genome sequence used for mapping DNA sequence data from sampled fish in this study can be found at https://www.ncbi.nlm.nih.gov/bioproject/PRJNA680301. The raw DNA sequence data from 40 individual fish and SNP array data from 1152 individual fish represent commercial IP owned by five Atlantic halibut producers, four in Norway and one in Scotland, all of which who have elected to remain anonymous in this study; these data are therefore not made available.The Affymetrics SNP array designed in this study is commercial IP of the company Cryogenetics AS who will gladly facilitate genotyping of halibut samples as a commercial service. Upon request, the genomic coordinates of all SNP loci present on the array may be obtained from Cryogenetics AS, Hamar, Norway.There are no phenotypes other than gender presented in this study and the genomic coordinates (based on the reference genome mentioned above) of 67 sex probes from the SNP array are listed in supplementary table S1 of this manuscript. Upon request, raw signal intensity data from these 67 probes on the array for the sexed fish may be obtained from Cryogenetics AS, Hamar, Norway.

## Abbreviations

CRAM: Compressed Reference-oriented Alignment Map
DNA: Deoxyribonucleic Acid
GS: Genomic Selection
gsdf: gonadal soma-derived factor
HWE: Hardy-Weinberg Equilibrium
LD: Linkage Disequilibrium
MAF: Minor Allele Frequency
MAS: Marker assisted selection
QC: Quality Control
SNP: Single Nucleotide Polymorphism
SV: Structural Variants
TE: Transposable Elements
VCF: Variant Call Format
WGS: Whole Genome Sequencing
