# Supplementary material for: Development and validation of a 70K SNP genotyping array for Atlantic halibut (Hippoglossus hippoglossus)
Source: BMC Genomics. 2025 Oct 16;26:924. doi: 10.1186/s12864-025-12128-1 (PMC12532428; doi:10.1186/s12864-025-12128-1)

**Supplementary materials**


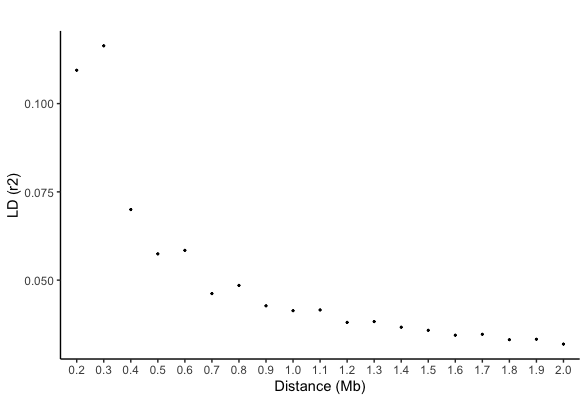


***Figure S1.*** *Decay of average LD (r^2^) over distance among SNPs in Atlantic halibut (Hippoglossus hippoglossus) population. The black dots show the average r^2^ within each bin, with a sliding window 20 Kb.*

***Table S1.*** *List of* *SNP probes tested for sex identification.* *Results showing binding sites of 67 oligonucleotides (individual sex probe sequences) on chromosome 13(LG13) of Atlantic halibut; fwd: forward.*

| **Primer name** | **Orientation** | **Region** |
| --- | --- | --- |
| AX-614943875 | fwd | 1949135..1949205 |
| AX-614944272 | fwd | 1949135..1949205 |
| AX-614945806 | fwd | 7831657..7831727 |
| AX-615035026 | fwd | 2038356..2038426 |
| AX-615035094 | fwd | 8319153..8319223 |
| AX-615035096 | fwd | 8336622..8336692 |
| AX-615035160 | fwd | 2038356..2038426 |
| AX-615035231 | fwd | 8336622..8336692 |
| AX-634289051 | fwd | 8503871..8503907 |
| AX-634289052 | fwd | 8503872..8503908 |
| AX-634289054 | fwd | 8503874..8503910 |
| AX-634289055 | fwd | 8503875..8503911 |
| AX-634289056 | fwd | 8503876..8503912 |
| AX-634289057 | fwd | 8503877..8503913 |
| AX-634289058 | fwd | 8503878..8503914 |
| AX-634289059 | fwd | 8503879..8503915 |
| AX-634289060 | fwd | 8503880..8503916 |
| AX-634289061 | fwd | 8503881..8503917 |
| AX-634289082 | fwd | 8509689..8509725 |
| AX-634289092 | fwd | 8505103..8505139 |
| AX-634289093 | fwd | 8505104..8505140 |
| AX-634289094 | fwd | 8505105..8505141 |
| AX-634289095 | fwd | 8505106..8505142 |
| AX-634289096 | fwd | 8505107..8505143 |
| AX-634289098 | fwd | 8505109..8505145 |
| AX-634289101 | fwd | 8505112..8505148 |
| AX-634289102 | fwd | 8505113..8505149 |
| AX-634289103 | fwd | 8505114..8505150 |
| AX-634289104 | fwd | 8505115..8505151 |
| AX-634289105 | fwd | 8505116..8505152 |
| AX-634289106 | fwd | 8505117..8505153 |
| AX-634289107 | fwd | 8505118..8505154 |
| AX-634289119 | fwd | 8509690..8509726 |
| AX-634289120 | fwd | 8509691..8509727 |
| AX-634289121 | fwd | 8509692..8509728 |
| AX-634289122 | fwd | 8509693..8509729 |
| AX-634289123 | fwd | 8509694..8509730 |
| AX-634289124 | fwd | 8509695..8509731 |
| AX-634289125 | fwd | 8509696..8509732 |
| AX-634289126 | fwd | 8509697..8509733 |
| AX-634289127 | fwd | 8509698..8509734 |
| AX-634289128 | fwd | 8509699..8509735 |
| AX-634289129 | fwd | 8509700..8509736 |
| AX-634289130 | fwd | 8509701..8509737 |
| AX-634289132 | fwd | 8509703..8509739 |
| AX-634289133 | fwd | 8509704..8509740 |
| AX-634289134 | fwd | 8509705..8509741 |
| AX-634289135 | fwd | 8509706..8509742 |
| AX-634289137 | fwd | 8509708..8509744 |
| AX-634289138 | fwd | 8509709..8509745 |
| AX-634289139 | fwd | 8509710..8509746 |
| AX-634289141 | fwd | 8509712..8509748 |
| AX-634289156 | fwd | 8509727..8509763 |
| AX-634289157 | fwd | 8509728..8509764 |
| AX-634289158 | fwd | 8509729..8509765 |
| AX-634289160 | fwd | 8509731..8509767 |
| AX-634350075 | fwd | 8509744..8509780 |
| AX-634350076 | fwd | 8509745..8509781 |
| AX-634350077 | fwd | 8509746..8509782 |
| AX-634350078 | fwd | 8509747..8509783 |
| AX-634350079 | fwd | 8509748..8509784 |
| AX-634350080 | fwd | 8509749..8509785 |
| AX-634350083 | fwd | 8509752..8509788 |
| AX-634350084 | fwd | 8509753..8509789 |
| AX-634350085 | fwd | 8509754..8509790 |
| AX-634350118 | fwd | 8509688..8509724 |
| AX-634350137 | fwd | 8509740..8509776 |

***Table S2.*** *List of SNP Parameters used in PLINK to run LD.*

| **Parameters used in PLINK to run LD** | |
| --- | --- |
| Missingness per SNP | --geno 0.1 |
| Missingness per individual | --mind 0.1 |
| Minor allele frequency | --maf 0.05 |
| Hardy-Weinberg Threshold | -- hwe 0.0000001 |
| Linkage disequilibrium | – ld-window-r2 0 |

**
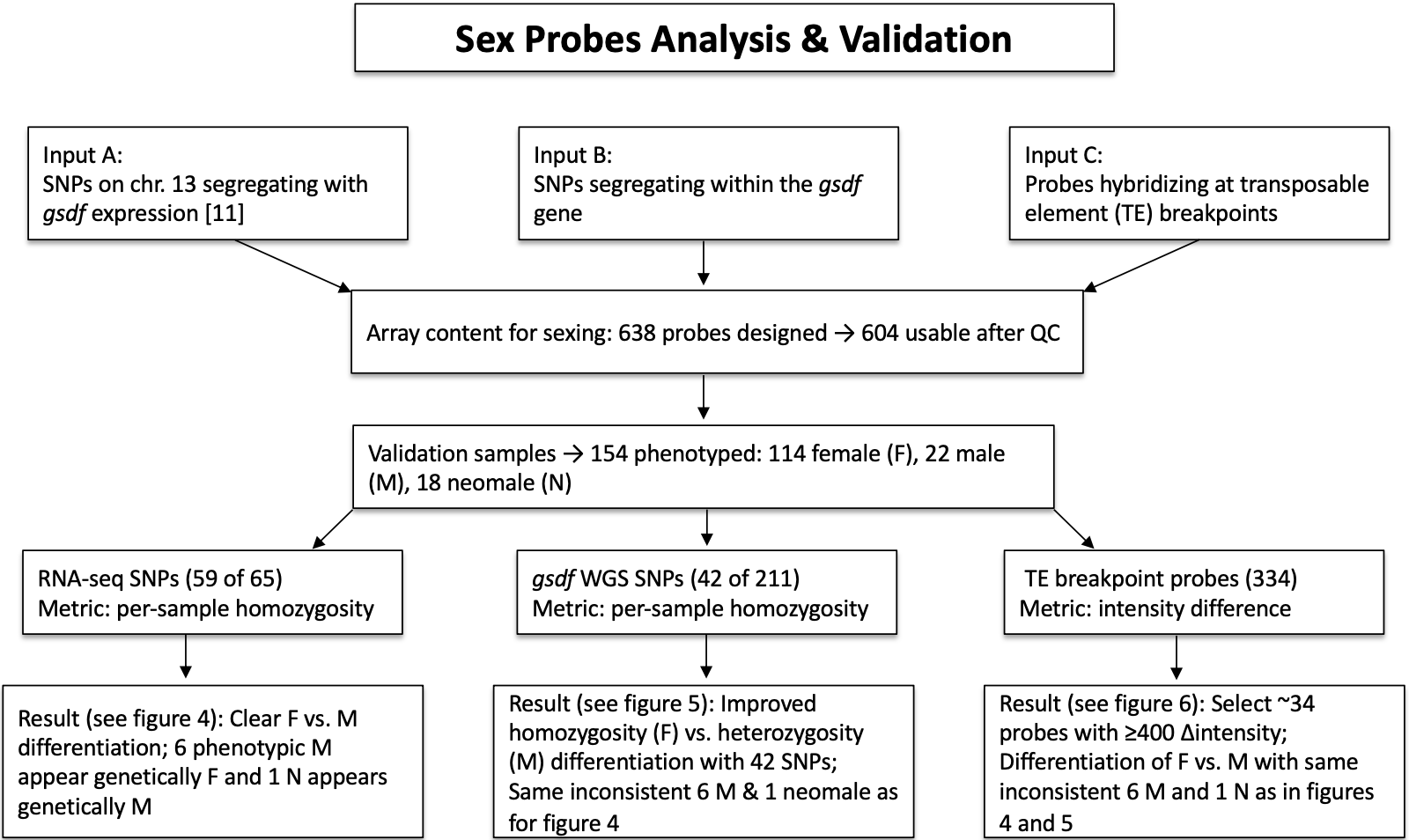
**

***Figure S2.*** *Diagram summarizing the workflow of the sex probes analysis and validation.*

***Figure S3.*** *Proportion of homozygosity for 211 SNPs across the gsdf region per individual. The trend is for males to display lower homozygosity, but the contrast is not strong.*
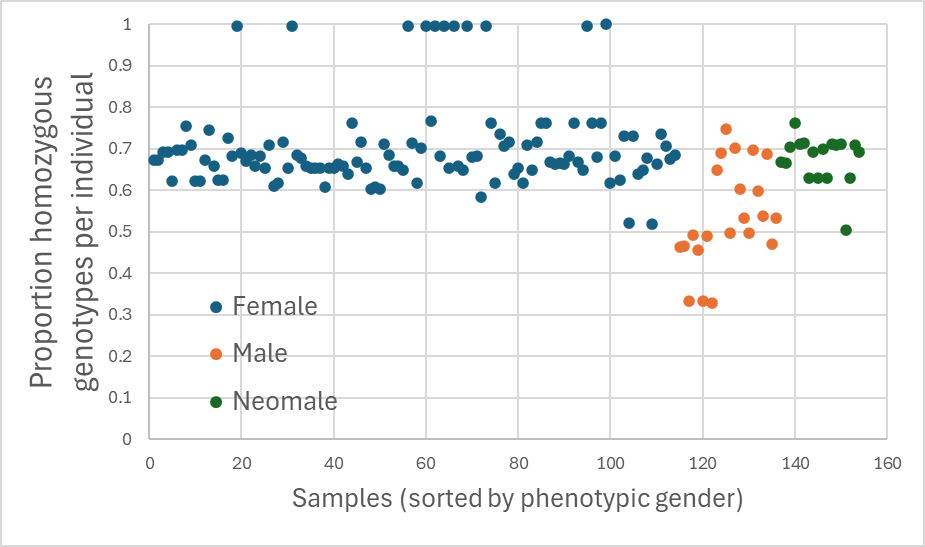

Supplement: Supplementary file 1 — Supplementary Material 1. Figure S1. Decay of average LD (r2) over distance among SNPs in Atlantic halibut (Hippoglossus hippoglossus) population. The black dots show the average r2 within each bin, with a sliding window 20 Kb. Table S1. List of SNP probes tested for sex identification. Results showing binding sites of 67 oligonucleotides (individual sex probe sequences) on chromosome 13(LG13) of Atlantic halibut; fwd: forward. Table S2. List of SNP Parameters used in PLINK to run LD. Figure S2. Diagram summarizing the workflow of the sex probes analysis and validation. Figure S3. Proportion of homozygosity for 211 SNPs across the gsdf region per individual. The trend is for males to display lower homozygosity, but the contrast is not strong. [file 12864_2025_12128_MOESM1_ESM.docx]
